# Prospecting Pharmacologically Active Biocompounds from the Amazon Rainforest: In Vitro Approaches, Mechanisms of Action Based on Chemical Structure, and Perspectives on Human Therapeutic Use

**DOI:** 10.3390/ph17111449

**Published:** 2024-10-30

**Authors:** Andryo O. de Almada-Vilhena, Oscar V. M. dos Santos, Milla de A. Machado, Cleusa Y. Nagamachi, Julio C. Pieczarka

**Affiliations:** Center for Advanced Biodiversity Studies, Cell Culture Laboratory, Institute of Biological Sciences, Federal University of Pará/Guamá Science and Technology Park, Avenida Perimetral da Ciência Km 01—Guamá, Belém 66075-750, PA, Brazil; andryoorfi@hotmail.com (A.O.d.A.-V.); oscar.vinicius.ms@gmail.com (O.V.M.d.S.); millaamachado@gmail.com (M.d.A.M.); cleusanagamachi@gmail.com (C.Y.N.)

**Keywords:** bioassay, human health, natural resources, phenolic compounds, terpenes

## Abstract

The Amazon rainforest is an important reservoir of biodiversity, offering vast potential for the discovery of new bioactive compounds from plants. In vitro studies allow for the investigation of biological processes and interventions in a controlled manner, making them fundamental for pharmacological and biotechnological research. These approaches are faster and less costly than in vivo studies, providing standardized conditions that enhance the reproducibility and precision of data. However, in vitro methods have limitations, including the inability to fully replicate the complexity of a living organism and the absence of a complete physiological context. Translating results to in vivo models is not always straightforward, due to differences in pharmacokinetics and biological interactions. In this context, the aim of this literature review is to assess the advantages and disadvantages of in vitro approaches in the search for new drugs from the Amazon, identifying the challenges and limitations associated with these methods and comparing them with in vivo testing. Thus, bioprospecting in the Amazon involves evaluating plant extracts through bioassays to investigate pharmacological, antimicrobial, and anticancer activities. Phenolic compounds and terpenes are frequently identified as the main bioactive agents, exhibiting antioxidant, anti-inflammatory, and antineoplastic activities. Chemical characterization, molecular modifications, and the development of delivery systems, such as nanoparticles, are highlighted to improve therapeutic efficacy. Therefore, the Amazon rainforest offers great potential for the discovery of new drugs; however, significant challenges, such as the standardization of extraction methods and the need for in vivo studies and clinical trials, must be overcome for these compounds to become viable medications.

## 1. Introduction

The Amazon rainforest holds a large part of the planet’s biodiversity. Regarding plants, it is estimated that there are around 14,000 species cataloged in the Amazon, and these individuals are excellent candidates for offering biomolecules and extracts of pharmacological and/or biotechnological interest [1], making the Amazon a huge storehouse of new biotechnologies, characterized as a “modern *Eldorado*” for bioprospecting [2]. Current estimates suggest there are around 40,000 plant species, including those still not catalogued by the scientific community [3].

According to a survey conducted by Verpoorte [4] via NAPRALERT (Natural Products Alert; linked to The Program for Collaborative Research in the Pharmaceutical Sciences, currently discontinued) in the 1990s, around 13,952 plant species had been identified in the world that exhibit potential pharmacological activity. Due to this potential, Liu et al. [5] propose a novel approach for classifying vegetable organisms, based on the similarity of synthesized metabolites, which would facilitate the direction of research and conditions of use of these biomolecules. It is interesting to highlight that, as a result of the aforementioned study, a matrix was generated that classifies plants into edible, medicinal, timber, decorative, and poisonous, and the methodology used was able to accurately separate edible and medicinal plants due to metabolomic differences intrinsic to each group.

In this sense, the use of natural products arbitrarily can cause harmful effects on the individual’s health. Therefore, studies are needed to look for traces of toxicity in biocompounds to determine safe use standards. Since the advent of toxicology as a scientific field, several in vitro and in vivo methodology protocols have been developed to predict unwanted effects, as well as to prove the effectiveness of the most varied plant compounds and standardize their uses.

In vitro biological assays gained notoriety in the 1980s when regulatory entities around the world began to worry about reducing the use of animals in testing. This test modality consists of using chemical reactions and/or parts of organisms (cell culture) in order to mimic, in laboratory conditions, biological processes that occur naturally or that can be induced [6]. In vivo insurability tests consist of the use of animals as test subjects (rats, rabbits, monkeys, fishes, among others), that is, complete organisms aiming to visualize global effects not predicted in in vitro tests.

Using both bioassay modalities (in vivo and in vitro), a range of alterable biological markers can be assessed through the induction of chemical agents: the assessment of oxidative stress; the investigation of antioxidant activity; the assessment of membrane integrity; the rates of DNA fragmentation and changes in the chromosomal set; the differential expression of genes linked to different metabolic pathways; the evaluation of cytotoxicity and acute and chronic toxicity; among others. The pharmaceutical and biotechnology industries use these types of methodologies in order to establish safe conditions for the use of the most diverse compounds [7,8,9,10].

Therefore, considering the pharmacological potential of the Amazon region, this work aims to review the literature about the use of bioassays to investigate the efficacy and safety of extracts obtained from Amazonian plants, identifying the most promising compounds as well as their biological activities. Additionally, it seeks to discuss the challenges and necessary steps for transforming Amazonian biocompounds into marketable medications, addressing issues such as standardization, clinical testing, and regulation. This work provides a valuable synthesis of current knowledge and state-of-the-art of in vitro prospection on the pharmacological potential of Amazonian biodiversity. It encourages researchers to pursue bioprospecting in the Amazon and develop new strategies for drug discovery, which could lead to sustainable and ethically sourced pharmaceutical products.

## 2. Exploration of Advantages and Disadvantages of In Vitro Approaches

In vitro studies are essential for pharmacological and biotechnological research, allowing scientists to investigate biochemical and biological processes and test interventions in a controlled and direct manner; however, these tests have advantages and disadvantages that must be considered before, during, and after the test is performed [11,12].

Conducting experiments in vitro is typically more expeditious and cost-effective compared to in vivo studies, which frequently necessitate the use of animal or human models and require a greater allocation of time and resources for execution and analysis. In vitro studies offer more controlled and standardized conditions, thereby enhancing the reproducibility of experiments. This control not only improves the precision of the data but also facilitates the comparison of results across different experimental conditions [12].

Another advantage of in vitro studies is the ability to precisely control experimental conditions [13]. In a laboratory environment, it is possible to adjust and monitor variables, such as temperature, pH, nutrient concentration, and the presence of external agents. This provides detailed control over the factors that can influence the results, allowing for a more precise analysis of the effects of a substance or treatment [12,14]. In vitro studies also allow for the investigation of effects in isolation, without the interference of the complex and variable systems of living organisms. This facilitates the identification of specific mechanisms of drug action or the understanding of the effects of genetic mutations in isolated cells, for example [12].

On the other hand, it is important to highlight the disadvantages of in vitro approaches. Firstly, these methods cannot replicate the full complexity of a living organism, as in vitro systems generally fail to mimic the intricate interactions between various types of cells, tissues, and organs that occur within a complete specimen [15,16,17]. Additionally, there is the absence of a physiological context, as cells or tissues cultured may not respond as they would in a complete physiological environment. For example, hormones, blood flow, and cellular interactions that influence biological responses in a living organism may be absent or insufficiently represented in a culture system [18].

**Figure 1:**
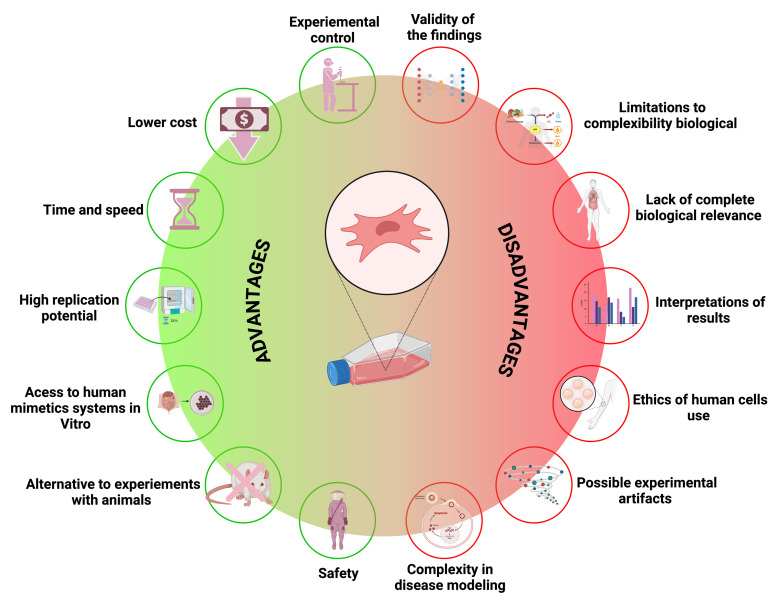
Comparison of the advantages and disadvantages of in vitro studies.

Furthermore, results obtained in vitro may not always translate directly to in vivo models, as the efficacy or toxicity of a substance observed in the cell culture may not exactly match what occurs in a living organism due to differences in pharmacokinetics and biological interactions; cells grown in vitro often have a limited lifespan and may undergo phenotypic or functional changes over time. Additionally, the artificial environment of a culture may induce responses that are not representative of normal conditions within an organism. Finally, although in vitro studies avoid some of the ethical issues associated with the use of animals, concerns remain about the representativeness of culture models in relation to real human conditions, and the need for the validation of these models with more complex systems still persists [12,19,20,21,22,23]. Figure 1 compares the advantages and disadvantages of in vitro techniques.

## 3. The Amazon Rainforest as a Source of Potential Natural Pharmaceuticals

The relationship between in vitro assays and Amazonian biodiversity is a fascinating example of how scientific research can interact with the sustainable development of natural resources, as the Amazon is a rich source of plants (and other organisms) with the potential to produce new bioactive compounds. In vitro assays, based on biochemical reactions or bioassays in cells or tissues outside the whole organism, can be used, in the first place, to test these biocompounds for pharmacological, antimicrobial, or anticancer activities, indicating the efficacy and safety of these products, allowing for the development of new therapies and applications based on natural resources from the Amazon.

Given the vast biodiversity of the Amazon, numerous research groups have explored the potential biological activities of plant extracts through in vitro methodologies. Table 1 provides a summary of the findings from the reviewed articles. These extracts, obtained using various techniques from a wide array of plant species, have been reported to possess multiple uses due to their biocompounds. To systematically assess these potential activities from Amazon vegetables, we conducted an extensive literature review focusing on studies that employed in vitro assays to evaluate the safety and efficacy of metabolites derived from species native to the Amazon region. The search strategy employed is detailed in the Supplementary Data.

Our analysis of the literature reveals a significant interest among research groups in investigating the biological activities of plant metabolites for various applications. Although there is no standardized preference for the plant parts used in extraction processes, it is evident that many studies are informed by the empirical knowledge of traditional communities. The chemical compounds most frequently derived from these plants include fatty acids, phenolic compounds, terpenes, and quinones.

## 4. Why Do Some Extracts Exhibit Multiple Pharmaceutical Properties?

Several factors can influence the biological activity of a plant extract, leading to significant variations in its efficacy, safety, and application. First, different species, subspecies, or varieties of a plant may have distinct chemical profiles, and the composition of secondary metabolites may vary depending on the plant’s developmental stage. Similarly, different parts of the plant—such as leaves, roots, seeds, bark, or flowers—may contain varying active compounds in differing quantities, resulting in diverse pharmacological properties [49,50,51,52].

The choice of solvent (e.g., water, ethanol, methanol, acetone, hexane) also plays a critical role in determining which compounds are extracted, as different chemicals have distinct solubilities. Other factors, such as temperature, extraction time, and pH, can affect the efficiency of the extraction process and the stability of bioactive compounds. Environmental factors, including temperature, humidity, sunlight exposure, and altitude, influence the chemical profile of plants, as they produce different secondary metabolites in response to environmental stress. Soil mineral composition and pH also impact nutrient availability, which can alter the production of bioactive compounds in plants. Furthermore, the season in which the plant is harvested can affect its chemical composition, especially for plants that produce secondary metabolites in response to seasonal changes [49,53,54,55,56].

During the processing of plant material (drying, grinding, and storage), the stability of bioactive compounds can be affected. Excessive heat or prolonged exposure to light and oxygen may degrade certain components. Additionally, factors such as temperature, humidity, and light exposure during the storage of extracts or raw plant materials can influence the stability and, consequently, the efficacy of bioactive compounds [57,58].

Furthermore, certain compounds within a plant extract may act synergistically, enhancing biological activity, while others may inhibit specific components (antagonism), reducing overall efficacy. Biological activity is often dose-dependent, with varying concentrations of bioactive compounds leading to different levels of therapeutic or toxic activity. Lastly, the biological activity of an extract can vary depending on the target organism or cell, producing distinct effects depending on the biological system under study (e.g., in vitro, in vivo, humans, animals, bacteria) [59,60,61].

## 5. Chemical Characterization of Phenolic Compounds

Phenolic compounds represent a broad class of organic molecules characterized by the presence of at least one benzene ring attached to hydroxyl (-OH) groups. These compounds are widely distributed in plants and are known for their significant biological activities, including antioxidant, anti-inflammatory, antimicrobial, and anticancer properties. Structurally, phenolic compounds are classified into various subgroups, such as phenolic acids, flavonoids, tannins, and lignans, each distinguished by unique structural features [62,63].

Phenolic acids are characterized by a benzene ring with one or more carboxyl (-COOH) and hydroxyl group substitutions. Flavonoids consist of a basic structure of 15 carbon atoms arranged into two aromatic rings (A and B), connected by a three-carbon chain that forms the heterocyclic C ring. Tannins are complex phenolic polymers, subdivided into hydrolysable and condensed tannins. Lignans are dimers of phenylpropanoid units, comprising two aromatic rings connected by a three-carbon chain [64,65].

These structural variations across phenolic subgroups directly influence their chemical and biological properties, making them a central focus in medicinal chemistry and phytochemistry. Furthermore, interactions between the functional groups in these different classes can enhance their health benefits, particularly in neutralizing free radicals and modulating inflammatory pathways [66,67,68].

### 5.1. Antioxidant, Anti-Inflammatory, and Antineoplastic Activity of Phenolic Compounds

Açaí (*Euterpe oleracea*), abundant in the Amazon rainforest, is widely recognized as an excellent source of anthocyanins, flavonoids, and polyphenols [69,70]. These antioxidants are essential in neutralizing free radicals in the body, particularly within cardiovascular tissues, thereby reducing chronic inflammation associated with cardiovascular diseases, such as atherosclerosis [71,72]. The neuroprotective effects of açaí are likely attributed to its ability to restore electron transport function in the mitochondrial chain of neuronal cells affected by mitochondrial complex I dysfunction, through the upregulation of transcriptional genes [73]. Additionally, due to its antioxidant properties, açaí also reduces L-glutamate levels in neural cells, a mechanism closely associated with neurodegenerative diseases [74].

The phenolic compounds in the pulp from fruits of *E. oleracea* exhibit significant antioxidant activity, demonstrating an ability to neutralize free radicals, which may play a crucial role in protecting against oxidative stress. Using the FRAP (ferric reducing antioxidant power) assay, researchers found that a substantial number of antioxidants remained active in the extract even after simulated digestion. These compounds also showed a notable reduction in DNA damage induced by hydrogen peroxide (H_2_O_2_), a known genotoxic agent [27].

The presence of multiple hydroxyl groups on the aromatic ring enhances the phenolic compound’s ability to donate hydrogens and stabilize free radicals. Ortho (adjacent position) or para (opposite position) hydroxyl groups relative to other substituents on the ring, as well as conjugation—the presence of an extended pi system alternating double and single bonds on the aromatic ring—facilitate the stabilization of free radicals formed after hydrogen donation, thereby increasing antioxidant capacity. Substituents such as methoxy groups (-OCH_3_) can also influence antioxidant activity by stabilizing the formed radicals or altering the electronic distribution within the molecule [75,76,77,78,79].

The polar extract of *Byrsonima crassifolia* (obtained with CO_2_ + ethanol) showed greater antioxidant activity, with ORAC (oxygen radical absorbance capacity) values reaching 122.61 µmol TE/g and DPPH at 17.14 µmol TE/g. In addition, this showed high levels of phenolic compounds (up to 20.63 mg GAE/g) and flavonoids (0.65 mg QE/g), both known for their significant antioxidant properties. Although it did not show cytotoxicity in HepG2 (human hepatoma) cells, the polar extract did show relevant cytoprotective effect under H_2_O_2_ stress in HepG2 cells [44].

In addition, the ethanolic extract of *Libidibia ferrea* exhibits notable antioxidant and antineoplastic activities. The antioxidant effects have been attributed to the presence of phenolic compounds, such as quinic acid and benzoic acid, which inhibit free radical formation. The antineoplastic potential was investigated in human gastric adenocarcinoma cells (ACP02), where the extract inhibited cell migration, likely due to cytoskeletal disorganization, thereby preventing the progression of metastasis [28,80].

Decoction extracts from the bark of *Couroupita guianensis* display notable anti-inflammatory and wound-healing activities. The main compound identified, a sulfate derivative of ellagic acid, a phenolic compound, stimulates the migration of human keratinocytes (HaCaT), promoting wound closure. The wound-healing mechanism involves the phosphorylation of the ERK1/2 and AKT pathways, as well as the increased expression of MMP2, which is crucial for extracellular matrix remodeling. Additionally, the inhibition of NF-κB activation confers anti-inflammatory effects [25,81,82].

The infused extract from the leaves of *Copaifera malmei* is notable for its ability to prevent DNA damage, providing protection against genetic damage induced by hydrogen peroxide (H_2_O_2_) in Chinese hamster ovary cells (CHO-k1) in both pre-exposure and cotreatment protocols. In cotreatment, the protective effect was observed only at the lowest concentration; whereas, postexposure treatment resulted in increased DNA damage, particularly at higher concentrations. The antigenotoxic mechanism of this extract is likely related to its ability to modulate the cellular antioxidant system, enhancing the activity of enzymes such as catalase and glutathione peroxidase, which play key roles in eliminating H_2_O_2_ and protecting against oxidative damage. Additionally, the extract demonstrated a reduction in myeloperoxidase activity, an enzyme that contributes to the generation of reactive oxygen species (ROS), suggesting that DNA protection may be associated with reduced oxidative stress [45].

Another common species in the Amazon region is *Astrocaryum aculeatum*, popularly known as “tucumã”. According to Cabral et al. [26], the primary compounds identified in the extract include ellagic acid, which exhibits anti-inflammatory activity and reduces the levels of ROS and nitric oxide (NO). This involves a decrease in the expression of cytokine-related genes such as IL-1β and IL-6 (interleukin), while increasing the expression of IL-10, thus enhancing antioxidant defenses (superoxide dismutase and catalase) and leading to a reduction in oxidative stress.

The presence and position of hydroxyl groups on the aromatic ring affect the compound’s ability to inhibit proinflammatory enzymes, such as cyclooxygenase (COX) and lipoxygenase (LOX). For instance, compounds with multiple ortho hydroxyl groups show a greater potential to neutralize ROS, which are mediators of inflammation [83,84,85]. Conjugation can stabilize the molecule and enhance its ability to interact with cellular receptors and enzymes involved in inflammatory responses [86,87]. The addition of methoxy groups or carboxyl groups (-COOH) to the phenolic structure can enhance its ability to cross cell membranes and reach molecular targets, such as nuclear receptors involved in the regulation of gene expression of inflammatory mediators. Certain phenolic compounds can interfere with the activation of transcription factors such as NF-κB (nuclear factor kappa-light-chain-enhancer of activated B cells) and AP-1 (activator protein-1), which regulate the expression of proinflammatory genes [87,88].

The phenolic compounds present in the ethanolic and hydroethanolic extracts of *Caryocar villosum* exhibit a high capacity to neutralize free radicals, particularly in ABTS (2,2’-Azino-bis(3-ethylbenzothiazoline-6-sulfonic acid)) and DPPH (2,2-Diphenyl-1-picrylhydrazyl) assays. The anti-inflammatory activity is promoted by the inhibition of nitric oxide production in J774 cells (murine macrophage), alongside antineoplastic effects on SKMEL19 (human melanoma), MCF-7 (human breast cancer), and HCT116 (human colorectal cancer) cell lines [24].

Gatea et al. [37] tested phenolic compounds extracted from *Portulaca oleracea* and *P. pilosa*, observing a cytostatic effect in colorectal cancer cells (Caco-2), with the *P. pilosa* extract proving more effective. Notably, extracts of *Portulaca* species also contain polysaccharides, which directly induce apoptosis in cancer cells by regulating the cellular pathways that control survival and death, aiding in tumor suppression and inhibiting angiogenesis [89,90].

Conjugation allows for electron delocalization, which increases the stability of the phenolic compound and enhances its ability to interact with molecular targets, potentially leading to cell cycle inhibition and DNA damage, both of which are essential for antineoplastic activity. On the other hand, the presence of methoxy, alkyl, or carboxyl groups can alter the compound’s affinity for specific cellular targets, such as hormonal receptors or enzymes involved in cell cycle regulation [91,92,93].

Considering their antineoplastic potential, phenolic compounds can inhibit the activation of Nrf2, which is involved in protecting against oxidative stress and contributing to therapy resistance in cancer cells. They can also suppress the PTEN/Akt/mTOR (phosphatase and tensin homolog/protein kinase B/mammalian target of rapamycin) pathway, reducing cell survival and promoting apoptosis in tumor cells, thereby slowing cancer proliferation and progression. Additionally, these compounds act as HDAC (histone deacetylase) inhibitors, affecting histone acetylation and regulating gene expression involved in the cell cycle and apoptosis, leading to tumor growth inhibition in various cancer models [94,95,96,97]. Despite promising in vitro findings, in vivo studies and human clinical trials investigating the antioxidant, anti-inflammatory, and antineoplastic activities of phenolic compounds remain limited. Phenolic compounds are widely used in alternative medicine, often included in nutraceuticals and dietary supplements due to their ability to prevent oxidative damage and reduce inflammation, typically supported by in vitro studies. However, preclinical in vivo studies and human clinical trials remain scarce and, in some cases, yield divergent results.

For instance, Wistar rats with arthritis that received oral treatments of hesperidin (50 mg/kg) and daidzein (20 mg/kg) (both phenolic compounds) for 21 days showed a significant reduction in TNF-α (tumor necrosis factor-alpha) levels, a key cytokine in chronic inflammation. The treatments also reduced joint elastase activity, indicating decreased neutrophil infiltration and joint inflammation. Hesperidin exhibited strong antioxidant capacity, lowering oxidative stress by neutralizing free radicals, as evidenced by reduced MDA (malondialdehyde) levels and increased plasma antioxidant capacity. Both substances also significantly lowered LDL-C (low-density lipoprotein cholesterol), VLDL-C (very-low-density lipoprotein cholesterol), and triglyceride levels, while increasing HDL-C (high-density lipoprotein cholesterol), suggesting an improved lipid profile and reduced risk of cardiovascular diseases associated with arthritis [98].

A study with C57BL/6J (normal) and db/db (diabetic) mice investigated the impact of ellagic acid on metabolic disorders induced by subclinical hypothyroidism (SCH). Mice were treated with methimazole (MMI) to induce SCH, elevating TSH (thyroid-stimulating hormone) levels without altering free T4. The mice were divided into control and treatment groups receiving ellagic acid (100 mg/kg/day), MMI (0.08 mg/kg/day), or both simultaneously. After 12 weeks, ellagic acid significantly reduced glucose levels in MMI-treated mice, both normal and db/db, suggesting improved glucose homeostasis, enhanced glucose tolerance, reversed SCH-induced adverse effects on glucose metabolism, and mitigated kidney injuries exacerbated by SCH in db/db mice by reducing tubular atrophy and epithelial loss [99].

A phase I clinical study evaluating the safety and tolerability of carvacrol (a phenolic compound) in healthy human individuals divided forty participants into two groups: one receiving 1 mg/kg/day and the other 2 mg/kg/day for a month. In the 1 mg/kg/day group, there was a significant reduction in calcium and hemoglobin levels, as well as an increase in creatine kinase (CPK). In the 2 mg/kg/day group, there was a decrease in HDL cholesterol and total bilirubin levels, along with a significant improvement in forced expiratory volume, suggesting a potential respiratory benefit [100].

A randomized, double-blind, placebo-controlled clinical trial investigated the effects of ellagic acid on glycemic status, insulin resistance, oxidative stress, and inflammatory factors in human patients with type 2 diabetes *mellitus*. In this study, 44 patients with type 2 diabetes *mellitus* were randomly divided into two groups (22 patients in each): one group received 180 mg/day of ellagic acid for 8 weeks, while the other received a placebo. Treatment with ellagic acid significantly reduced glucose, HbA1c (glycated hemoglobin), insulin resistance, and triglyceride levels compared to the placebo group. There was also a significant increase in antioxidant enzyme activities (glutathione peroxidase and superoxide dismutase) and a decrease in oxidative stress markers (MDA) and inflammatory markers (TNF-α, IL-6, and C-reactive protein) in the ellagic-acid-treated group, suggesting that ellagic acid may be a useful dietary supplement to improve glycemic control and reduce chronic adverse effects in patients with type 2 diabetes [101].

Another randomized, double-blind, placebo-controlled clinical trial investigated the effects of quercetin supplementation on inflammatory factors and clinical symptoms of rheumatoid arthritis. Fifty women with arthritis were randomly divided into two groups: one group received 500 mg/day of quercetin for 8 weeks, while the other received a placebo. Inflammatory markers, such as high-sensitivity tumor necrosis factor (hs-TNF-α) and erythrocyte sedimentation rate, were assessed, along with clinical symptoms, such as morning stiffness, pain, and tender and swollen joint counts. Quercetin exhibited anti-inflammatory properties by inhibiting the production of inflammatory cytokines, including TNF-α, and modulating inflammatory signaling pathways, such as NF-κB activation. As a result, quercetin supplementation significantly reduced hs-TNF-α levels, morning stiffness, postactivity pain, and disease activity scores and health assessment questionnaire in the quercetin group compared to the placebo group. Although clinical symptoms improved significantly, no significant differences were observed in swollen or tender joint counts between the groups [102].

Currently, no isolated phenolic compound is directly used as a prescription anti-inflammatory drug or chemotherapeutic agent in humans. However, certain phenolic compounds, such as curcumin and resveratrol, have been extensively studied as adjuvants in anticancer therapies due to their antiproliferative effects [103,104]. Although the potential benefits of antioxidants are promising, the low bioavailability of some of these compounds limits their clinical efficacy, highlighting the need for strategies to enhance absorption [105].

### 5.2. Microbiological and Antiparasitic Activity of Phenolic Compounds

Phenolic compounds contain hydroxyl groups that can interact with bacterial membranes, destabilizing the lipid bilayer. This interaction affects membrane fluidity and permeability, facilitating the influx or leakage of essential cellular components, ultimately leading to cell death. Phenolics may denature or inactivate transmembrane proteins critical for vital functions, such as nutrient transport, signaling, and energy production (electron transport chain). Additionally, they may induce pore formation in the plasma membrane, allowing the leakage of intracellular materials, such as ions, ATP (adenosine triphosphate), and amino acids [67,68,106,107,108].

Furthermore, the chemical structure of the compound also influences its ability to cross cell membranes and its affinity for molecular targets, which are often modulated by the presence of specific functional groups, such as methoxy or carboxyl groups, that favor binding to specific enzymes or receptors in the parasites. The presence of conjugated systems, with alternating double bonds in the aromatic ring, facilitates the stabilization of free radicals generated during interactions with parasitic biomolecules necessary for the natural survival of the parasite, resulting in oxidative damage to lipids, proteins, and nucleic acids, thereby leading to growth inhibition or cell death [109,110,111,112].

The roots of *Deguelia nitidula* are a natural source of metabolites with antiparasitic and antibacterial activity. Ethanol extracts and their fractions exhibited significant activity against *Staphylococcus aureus*, demonstrating high selectivity and antibacterial efficacy [33]. Extracts of *Ambelania duckei* and *Curarea toxicofera* showed inhibition against epimastigote forms of *Trypanosoma cruzi*, with *C. toxicofera* being particularly effective, displaying an IC_50_ of 50 ± 5 µg/mL, while *A. duckei* had an IC_50_ of 221 ± 29 µg/mL. In comparison, the active control, benznidazole, had an IC_50_ of 0.7 µg/mL. None of the tested extracts exhibited cytotoxicity in HepG2 (liver adenocarcinoma) or MRC-5 (lung fibroblasts), indicating potential safety for future tests [34].

Phenolic compounds identified in *Equisetum hyemale* [32] (luteolin, coumarin, and rutin) demonstrated significant antimicrobial activity against various bacterial strains, including *Staphylococcus aureus*, *Escherichia coli*, *Pseudomonas aeruginosa*, and *Klebsiella pneumoniae*, suggesting promise as an alternative treatment for infections caused by micro-organisms resistant to synthetic drugs. The antimicrobial action of luteolin includes the inhibition of protein and peptidoglycan synthesis, as well as the modification of microbial membrane permeability [113,114]. As for coumarin, its antimicrobial activity is attributed to its lipophilic chemical structure, which facilitates penetration into microbial cells due to the presence of hydroxyl groups and the carbon chain length. It also interferes with biofilm formation by disrupting bacterial quorum sensing [115,116]. The rutin molecules act by altering membrane permeability, interfering with the production of essential metabolites for microbial survival, and reducing the transcription of the lasI, lasR, rhlI, rhlR, and pqsA genes [117]. The extracts and compounds showed no cytotoxicity or genotoxicity, suggesting that *E. hyemale* can be safely used in treating infections [32].

The crude ethanolic extract of *Eugenia stipitata* seeds demonstrated greater efficacy in inhibiting the development of gastrointestinal nematodes, as well as the free-living nematode *Panagrellus redivivus*, compared to dichloromethane and hydroalcoholic fractions. The tannins present in the seeds of *E. stipitata* are likely the main contributors to the observed antiparasitic activity, as these compounds have been associated with the inhibition of parasite fertility, egg hatching, and motility [31].

The polyphenols present in açaí (*E. oleracea*) may influence the gut microbiota and exhibit selective antimicrobial activities. Digestion of açaí polyphenols has been shown to impact the growth of certain bacterial groups, notably resulting in the significant inhibition of *Clostridium histolyticum* and the Bacteroides–Prevotella group. These polyphenols may inhibit the growth of harmful bacteria, while promoting the production of short-chain fatty acids, which provide health benefits to the host. Therefore, the consumption of açaí may act as a prebiotic, promoting the growth of beneficial bacteria and inhibiting pathogenic organisms. This suggests that the phenolic compounds in açaí possess antimicrobial properties that contribute to gut health [27].

Natural products often contain a complex mixture of compounds, which can complicate the identification and isolation of the specific component responsible for the desired antibiotic activity. This complexity also poses challenges in standardizing compositions to ensure consistent therapeutic effects, a critical requirement for antimicrobial drug approval.

## 6. Chemical Characterization of Terpenes

Terpenes are a diverse and complex class of organic compounds that play a central role in numerous biological functions. Structurally, they are derived from the union of isoprene units, which are composed of five carbon atoms and follow the general formula C_5_H_8_. The basic structure of terpenes, known as the “isoprene unit”, can repeat in various combinations, leading to the formation of different terpene subclasses, such as monoterpenes, sesquiterpenes, diterpenes, triterpenes, and polyterpenes [118,119,120].

Monoterpenes (C_10_) consist of two isoprene units and have ten carbon atoms. Sesquiterpenes (C_15_), with three isoprene units, contain fifteen carbon atoms. Diterpenes (C_20_) are composed of four isoprene units, while triterpenes (C_30_), which are built from six isoprene units, often form tetracyclic and pentacyclic structures. Finally, polyterpenes (C_40_ or more) are made up of more than eight isoprene units. Beyond their basic structural organization, terpenes are frequently modified through oxidation or enzymatic reactions, leading to the formation of terpenoids, which possess a wide range of bioactive properties. The structural versatility of terpenes enables them to participate in numerous biological and therapeutic processes, making them molecules of significant interest in pharmacology and biotechnology [120,121,122].

### Antioxidant, Anti-Inflammatory, Antineoplastic, and Antiparasitic Activity of Terpenes

Terpenes have demonstrated significant potential in reducing inflammation by decreasing the production of proinflammatory cytokines, such as TNF-α, IL-1, and IL-6, and inhibiting pathways, such as NF-κB, which are critical in the inflammatory response [123,124]. Terpenes have been particularly effective in treating neuroinflammatory conditions, such as Alzheimer’s and Parkinson’s diseases, by suppressing microglial activation, reducing oxidative stress, and downregulating inflammatory cytokines [125]. The spatial arrangement of atoms in a terpene molecule can influence its ability to interact with free radicals. For instance, terpenes with cyclic or branched structures may have greater flexibility or accessibility to interact with ROS, facilitating their neutralization. They may also contain functional groups, such as hydroxyls, which are important for the neutralization of free radicals [126,127,128,129].

Many terpenes exhibit cyclic structures, such as monoterpenes and sesquiterpenes, which can bind to specific membrane receptors or interact with cellular signaling pathways involved in the inflammatory response. The structural rigidity can increase the binding affinity with certain target proteins, modulating the expression of proinflammatory cytokines. The three-dimensional configuration of terpenes can influence their ability to activate or inhibit signaling pathways that regulate inflammation, such as the MAPK (mitogen-activated protein kinase) pathway. Additionally, the nonpolar nature of terpenes facilitates their interaction with lipid-rich cell membranes, allowing them to act directly at sites of inflammation, modulating intracellular processes, such as the inhibition of activation of proinflammatory transcription factors, such as NF-κB [130].

The methanolic and ethyl acetate extracts of *Curcuma kwangsiensis* (native from Asia) have shown significant anti-inflammatory activities, with the ethyl acetate extract yielding results comparable to aspirin in a carrageenan-induced rat edema model. The primary active compounds identified were sesquiterpenoids, which inhibited the production of inflammatory mediators, such as COX-2, IL-1β, and TNF-α. These extracts were also effective in reducing pain in an acetic-acid-induced writhing model, demonstrating a significant analgesic effect comparable to conventional painkillers [131]

D-limonene, a monoterpene, is well-known for its antioxidant, antitumor, and antidiabetic properties, among others. It regulates lipid metabolism by inhibiting adipocyte differentiation and promoting apoptosis of mature adipocytes. Additionally, d-limonene has been shown to reduce fatty acid synthesis, contributing to decreased fat accumulation in the liver. A randomized, double-blind, placebo-controlled study involving 60 patients over 12 weeks of d-limonene capsule administration concluded that d-limonene can regulate lipid metabolism and improve liver fat infiltration in overweight/obese patients. This suggests its potential as a safe and effective treatment for metabolic fatty liver disease [132].

In the Amazon context, andiroba (*Carapa guianensis*) is widely used in traditional medicine, and its oil contains a complex mixture of bioactive compounds [133], including antioxidant terpenes capable of inhibiting the production of proinflammatory mediators, such as nitric oxide (NO) and prostaglandins [119,134,135,136]. The major components of andiroba oil, primarily fatty acids and terpenes, are involved in modulating immune responses by regulating the activity of immune cells, particularly macrophages, which play a crucial role in inflammation. Besides its direct anti-inflammatory effects, andiroba oil is also known for its analgesic properties, reducing pain associated with inflammation. This analgesic effect is likely due to both the suppression of the inflammatory process and the potential inhibition of pain signal transmission [137,138,139].

Silica gel column chromatography has been employed to isolate bioactive compounds, such as carapanosins A, B, and C (terpenoids), from the seeds of *C. guianensis*, which exhibit strong anti-inflammatory activity. The anti-inflammatory mechanism of these compounds likely involves the modulation of the NO pathway and the regulation of inflammatory responses, contributing to reduced inflammation and enhanced healing [29].

Modifications in isoprene skeletons, such as the formation of terpenoids, can enhance antioxidant activity. Oxygenation, for instance, introduces heteroatoms that can participate in redox reactions, thereby increasing the antioxidant effect. Furthermore, as many terpenes are nonpolar and lipophilic, they are effective in protecting lipid-based cell membranes against lipid peroxidation, since their structure facilitates penetration into the cell membrane, where they can directly react with free radicals [140]. Terpenes that contain specific functional groups, such as hydroxyls, carbonyls, or epoxides, can interact with enzymes and receptors involved in inflammation, inhibiting the synthesis of inflammatory mediators, such as prostaglandins and leukotrienes, thereby resulting in anti-inflammatory effects [141].

Jerônimo et al. [39] reported the antineoplastic activity of terpene-rich extracts from seven species of the Myrtaceae family against various cancer cell lines, including breast (MCF7), colon (HCT116), stomach (AGP01), and melanoma (SKMEL-19) cells. Similarly, Da Silva et al. [40] investigated the essential oils of four *Eugenia* species, rich in sesquiterpenes, and reported inhibitory activity against colon cancer cells (HCT-116), though with high cytotoxicity observed in lung cells (MRC-5).

The shape and rigidity of the cyclic or linear structures of terpenes can determine their binding affinity to target proteins, such as enzymes and receptors that regulate the cell cycle and apoptosis. For example, terpenes with specific configurations can bind to the active sites of enzymes, such as topoisomerase (TOPO), inhibiting DNA replication in cancer cells. Lipophilic terpenes can easily be incorporated into cell membranes, influencing membrane fluidity and cellular signaling. This interaction can facilitate the entry of the terpene into the cell and promote the modulation of intracellular pathways that control cell proliferation and death, such as the PI3K/Akt/mTOR and MAPK signaling pathways [142,143,144,145].

The terpene transdehydrocrotonin, obtained via Soxhlet extraction from *Croton cajucara* (sacaca), demonstrates significant pharmaceutical activity, though hepatotoxicity has been reported. Molecular modifications carried out by Carvalho et al. [30] included synthesizing a carboxylated derivative from transdehydrocrotonin to reduce its toxicity, yielding positive effects. The mechanism of action involves inhibiting cell proliferation (HepG2) and inducing necrosis, with reduced genotoxicity compared to the original compound, indicating potential for therapeutic development with lower toxicity.

In addition to molecular modifications, nanoscale delivery systems can be employed to enhance the efficacy of such compounds. For instance, Chura et al. [41] studied the effects of essential oil from *C. cajucara*, rich in terpenes, and its encapsulation in nanostructured lipid carriers (NLCs) on A549 (human lung cancer cells) and BEAS-2B (normal human bronchial epithelial cells). The study revealed that A549 cancer cells were more resistant to the essential oil than the normal BEAS-2B cells. Nanostructured lipid carriers are encapsulation systems designed to deliver compounds such as drugs, nutrients, or bioactive substances. This technology enhances the solubility and stability of the compounds, enabling controlled and efficient administration by targeting specific tissues or cells, such as cancer cells, through surface modifications that facilitate selective interaction and sustained release [146,147,148,149].

Terpenes have been shown to induce apoptosis in tumor cells by activating proapoptotic signaling pathways, such as the caspase cascade, and regulating Bcl-2 (B-cell lymphoma 2) proteins, which control apoptosis [150,151]. Ursolic acid, for instance, has demonstrated the ability to inhibit angiogenesis by reducing the expression of proangiogenic factors, such as vascular endothelial growth factor (VEGF) [152,153].

Many terpenes also exhibit antioxidant activities that selectively modulate oxidative stress in tumor cells. For example, α-pinene protects normal cells from oxidative damage, while promoting the production of ROS in cancer cells, leading to selective tumor cell damage. Additionally, β-caryophyllene inhibits tumor cell invasion and metastasis by blocking the degradation of the extracellular matrix by matrix metalloproteinases, which are crucial for cancer cell migration and invasion into other tissues [154,155,156].

Other terpenes, such as betulinic acid (a triterpene) extracted from the bark of *Betula* species, are being investigated as potential chemotherapeutic agents for melanoma, lung cancer, and neuroblastoma [157,158,159]. Thapsigargin, a terpenoid found in *Thapsia garganica*, is another promising candidate for antineoplastic treatments, including prostate cancer [160]. Paclitaxel (Taxol), a diterpenoid extracted from the bark of *Taxus brevifolia* (Pacific yew), is widely used as a chemotherapeutic agent in treating various cancers, including breast, lung, and ovarian cancer [161,162]. Paclitaxel disrupts cell division by stabilizing microtubules during mitosis [163]. Another diterpenoid, docetaxel, is used to treat cancers such as prostate, breast, and lung [164].

Several terpenes are already in clinical use, not only for cancer treatment but also as antiparasitics [161,162,163,164]. Artemisinin, a sesquiterpene extracted from *Artemisia annua* (sweet wormwood), is primarily known for its use in treating protozoal infections, particularly malaria. Discovered by Nobel laureate Tu Youyou, artemisinin has demonstrated high efficacy against *Plasmodium falciparum*. Beyond its antimalarial use, studies have shown that artemisinin and its derivatives also possess antitumor activity [165,166,167].

Da Silva et al. [35] investigated the essential oils of three species of the genus *Ocotea* (native to the Amazon) for their chemical diversity and biological activity. The oils from *O. caudata*, *O. cujumary*, and *O. caniculata* are rich in sesquiterpenes, which exhibit antimicrobial activity against *Escherichia coli* and inhibitory activity against breast cancer cells (MCF-7). Sesquiterpenes can interact with cell membranes and disrupt the expression of genes involved in biofilm formation in micro-organisms, ultimately leading to cell death [168]. These compounds also interfere with the synthesis of the cell wall in both bacteria and fungi, weakening their defense mechanisms and making them more vulnerable to osmotic stress [169].

In this context, *Copaifera reticulata*, a plant species that produces resin rich in sesquiterpenes, has demonstrated low cytotoxicity in human fibroblasts (Wi 26VA-4) and likely exhibits antileishmanial activity, even against chloroquine-resistant strains (W2) [36]. The oleoresin of *C. reticulata* contains bioactive compounds that act directly against parasites of the *Leishmania* genus, disrupting the parasites’ cellular membranes, causing structural damage, and impairing their vital functions.

The nonpolar and lipophilic nature of terpenes facilitates their incorporation into the cell membranes of parasites, causing direct membrane damage, increasing permeability, and leading to cell rupture. Terpenes that contain reactive functional groups, such as epoxides, hydroxyls, and carbonyls, can form covalent bonds or interact with enzymes essential for the parasite’s survival, inhibiting the function of critical enzymes, such as proteases and reductases, which play key roles in the parasite’s metabolism. The shape and structural rigidity of terpenes, especially those with cyclic structures, can facilitate binding to specific receptors or ion channels present in parasites, negatively modulating signaling pathways essential for the parasite’s growth and reproduction, as well as affecting the transport of nutrients and ions [170,171,172,173,174].

## 7. Other Molecules with Pharmaceutical Activity: Fatty Acids and Quinones

Fatty acids are carboxylic acids characterized by long hydrocarbon chains that terminate with a carboxyl group. Chemically, fatty acids can be classified into several categories based on the structure of their hydrocarbon chains and the presence or absence of double bonds. The chemical structure of fatty acids is critical to their biological functions, including participation in cellular processes such as energy metabolism, cell signaling, and the formation of cellular membranes [175].

Unsaturated fatty acids in the *cis* configuration have a bent structure, which makes them more flexible and allows for better incorporation into cell membranes, positively regulating membrane fluidity, cell signaling, and the response to oxidative stress—important factors in cancer prevention. Furthermore, this conformation enhances interactions with other molecules, such as proteins and antioxidant enzymes, improving the ability to react with free radicals and aiding in protection against oxidative stress [176,177,178,179].

Trans fatty acids, on the other hand, have a more linear structure, and the arrangement of trans double bonds makes them less efficient at incorporating into cell membranes and modulating anti-inflammatory pathways. This characteristic imparts proinflammatory effects to this class of fatty acids, which are associated with an increased risk of cancer, as they can negatively affect the integrity of cell membranes and promote inflammatory processes, thereby increasing oxidative stress [180,181].

The oil extracted from andiroba (*C. guianensis*) contains fatty acids, predominantly oleic acid, palmitic acid, and linoleic acid, in addition to sterols, such as squalene, and triterpenic compounds, such as epoxygodunin and deacetylgedunin. This oil exhibits cytotoxicity in gastric adenocarcinoma cells (ACP02), which indicates its potential therapeutic use in cancer treatments without causing significant mutagenicity. The apoptosis is mediated by the fatty acids present in the oil, which can induce alterations in cell membranes and activate pathways of programmed cell death [38].

In a study conducted by Araújo-Lima et al. [46], the oil from andiroba demonstrated significant antioxidant activity and cytotoxicity in eukaryotic cells (CHO-K1 and RAW264.7). The antioxidant activity was more pronounced in oil extracted without heating, suggesting that thermal processing may degrade bioactive compounds, such as phenolics, which are responsible for scavenging free radicals. The oils also exhibited cytotoxic activity, attributed to the presence of terpenes with biological properties, including antifungal, bactericidal, and anti-inflammatory effects. Oil extracted without heating did not show significant mutagenic or genotoxic effects, making it a safer option compared to oils extracted using the autoclave and Soxhlet methods, which induced DNA damage and micronuclei formation in cells. This DNA damage was linked to the increased temperature during extraction, which seems to elevate the presence of compounds capable of inducing genotoxic effects.

The nanoemulsion of andiroba oil exhibited concentration-dependent cytotoxicity in NIH/3T3 cell line (mouse fibroblasts), although with a lower toxicity compared to crude oil. Encapsulation in nanostructures reduced toxicity and increased biological efficacy. The penetration capability and controlled release of bioactive compounds were enhanced due to the reduced particle size in the nanoemulsion. In in vivo tests on rats, the andiroba nanoemulsion showed no genotoxicity, cytotoxicity, or hematotoxicity. These results suggest that nanostructured andiroba oil could be a promising tool for future cosmetic or pharmaceutical applications, with potential uses as an anti-inflammatory agent and in skin care [48].

The murici (*Byrsonima crassifolia*) oil (obtained with supercritical CO_2_) showed high levels of lutein (up to 224.77 µg/g) and unsaturated fatty acids, such as oleic, linoleic, and palmitic acid. Although its antioxidant activity was lower than that of the polar extract, with ORAC of 43.48 µmol TE/g and DPPH of 6.04 µmol TE/g, the oil stood out for its cytoprotective effect. Furthermore, the nonpolar extract did not demonstrate cytotoxicity, qualifying it as a promising candidate for applications in food, cosmetics, and pharmaceuticals [44].

Açaí oil is composed of a variety of fatty acids, the most prominent being oleic, palmitic, γ-linolenic, linoleic, and palmitoleic acids, along with phenolic acids (vanillic, caffeic, ferulic, and cinnamic) and flavonoids (quercetin and kaempferol). In cell viability assays, açaí oil did not exhibit significant cytotoxicity at concentrations up to 1000 µg/mL. Regarding genotoxicity, comet and micronucleus assays revealed that the oil did not cause significant DNA damage in human cells. Moreover, açaí oil did not show chemoprotective effects against DNA-damaging agents, such as methyl methanesulfonate and benzo[a]pyrene [42].

Some clinical trials have reported that compounds from açaí improved antioxidant markers without affecting glycemia or lipid profiles. These studies observed increases in ApoA-I (apolipoprotein A-I) and total antioxidant capacity without influencing glucose, insulin, or cholesterol levels, improved vascular function without impacting heart rate or glycemia, and enhanced exercise endurance in athletes. Additionally, capsules lowered systolic blood pressure within 6 h, reduced tinnitus discomfort, improved anxiety symptoms, and lowered oxidative stress, while improving inflammatory markers in patients with metabolic syndrome [182,183].

An ongoing clinical trial is investigating the effect of andiroba gel on reducing dental sensitivity after teeth whitening. Participants are divided into three groups: one receives the andiroba gel, another a potassium nitrate gel, and the third a placebo. All undergo teeth whitening with 35% hydrogen peroxide. Sensitivity is measured using a visual analog scale, while tooth color is assessed before and after the treatment [184].

Quinones are a class of organic compounds characterized by a distinctive chemical structure consisting of a conjugated aromatic ring containing two ketone groups (-C=O). The basic structural formula can be represented by a benzene ring where two double bonds (C=C) in the ring are replaced by carbonyl (C=O) bonds, creating a conjugated carbonyl system. Quinones can be easily reduced to hydroquinones, their fully hydrogenated forms. This equilibrium between quinone and hydroquinone is crucial in biochemical processes, such as cellular respiration and photosynthesis, where they act as electron carriers in mitochondrial electron transport chains [185,186,187].

The spatial configuration of quinone determines its affinity and mode of interaction with enzymes crucial for cell maintenance and survival. For instance, some quinones inhibit TOPO II, an essential enzyme for DNA unwinding during replication and transcription. The three-dimensional conformation can also enable these molecules to bind to specific sites on proteins that regulate the cell cycle, modulating pathways that control the proliferation and survival of tumor cells [188,189,190,191]. The three-dimensional structure influences the localization and efficiency of interactions, as quinones that accumulate in the mitochondria, for example, can promote the release of cytochrome C and activate intrinsic apoptotic pathways [192,193,194].

However, quinones are also used as medicinal drugs and chemotherapeutic agents. Notable examples include doxorubicin, a chemotherapy drug used to treat various cancers, including breast cancer, leukemia, and lymphomas. Doxorubicin works by intercalating DNA, inhibiting nucleic acid synthesis, and inducing apoptosis in cancer cells [195,196]. Coenzyme Q10 (CoQ10), also known as ubiquinone, is a naturally occurring antioxidant found in nearly every cell in the human body, particularly in energy-demanding organs such as the heart, liver, and kidneys. CoQ10 plays a key role in ATP production, the molecule responsible for providing energy for cellular processes. Additionally, CoQ10 acts as an antioxidant, protecting cells from oxidative stress and damage caused by free radicals, which can contribute to aging and various diseases. CoQ10 is generally considered safe, with few side effects; although, some individuals may experience mild gastrointestinal symptoms, such as nausea or diarrhea, especially at higher doses [197,198].

The presence of different functional groups in quinones can influence the ease with which they participate in redox reactions. Quinones with substituent groups that favor the stabilization of the reduced form (hydroquinone) tend to have greater antioxidant potential. Natural quinones, such as ubiquinone, have a long lipophilic tail that facilitates their incorporation into cell membranes, where they perform essential antioxidant functions, protecting cells against oxidative damage [78,199,200].

Isoeleutherin and eleutherin, naphthoquinones extracted from the plant *Eleutherine plicata*, have shown genotoxic and cytotoxic activity in various test systems, such as the *Allium cepa* model and HepG2 cells. These compounds interact with the enzyme TOPO II, which is critical in the DNA replication process. The inhibition of this enzyme by isoeleutherin and eleutherin results in the blocking of cell division, leading to programmed cell death (apoptosis). Molecular docking and dynamics studies have shown that both compounds form stable complexes with TOPO II, with eleutherin displaying slightly higher affinity for the enzyme, which may explain its greater genotoxicity [43].

Structural modifications in quinones, such as the addition of substituent groups, can influence their redox behavior, solubility, and selectivity for cancer cells. These modifications can optimize the three-dimensional shape to increase the affinity with specific targets in tumor cells. For example, mitomycin C is an antineoplastic quinone that, in addition to its redox activity, can form covalent bonds with DNA, inhibiting its replication in cancer cells. Its efficacy is closely related to the three-dimensional shape that allows this specific interaction with DNA [201,202,203,204].

Another study on *E. plicata* focused on evaluating the cytotoxicity, genotoxicity, and oral toxicity of ethanol extracts, the dichloromethane fraction, and the isolated compound isoeleutherin. Dichloromethane fraction and isoeleutherin exhibited higher cytotoxicity in HepG2 cells. The comet assay revealed that both dichloromethane fraction and isoeleutherin induced high levels of DNA damage in HepG2 cells, particularly at higher concentrations. Dichloromethane fraction demonstrated greater genotoxic potential compared to the ethanol extract, likely due to the higher naphthoquinone content in the dichloromethane fraction. Molecular docking studies indicated that isoeleutherin, eleutherin, and eleutherol bind to caspase-8, a crucial enzyme in the extrinsic pathway of apoptosis. Caspase-8 activation leads to the activation of executioner caspases (caspase-3, caspase-7), resulting in programmed cell death. In acute and subacute toxicity assays in mice, neither ethanol extracts nor dichloromethane fraction caused significant clinical changes or affected animal body weight. No relevant hematological or biochemical changes were observed, indicating that under the study conditions, these extracts have low toxic potential [47].

The planar structure of quinones facilitates electron delocalization, enhancing their ability to stabilize free radicals and act as antioxidants. Quinones with a specific structure can better fit into the active sites of antioxidant enzymes, increasing the efficiency of free radical neutralization. Additionally, structural modifications, such as the addition of side groups, can alter the redox behavior of quinones and, consequently, their antioxidant properties [199,205,206,207].

A study investigated the safety and pharmacokinetics of MB12066 (quinone), a β-lapachone derivative, in two placebo-controlled, double-blind clinical trials with single and multiple ascending doses in healthy individuals. MB12066 was well tolerated, with mild adverse events, such as diarrhea and abdominal pain, particularly at higher doses (300–400 mg). MB12066 acts on NAD(P)H quinone oxidoreductase 1, an enzyme important for cellular redox balance and protection against oxidative stress, showing potential for treating obesity and metabolic syndrome due to its effects on cellular energy metabolism [208].

Another clinical study on quinones, specifically focused on pyrroloquinoline quinone, examined its effects on stress, fatigue, sleep, and overall quality of life in workers. There was a notable reduction in stress and fatigue levels, as measured by the Profile of Mood States. Quality of life scores, particularly related to appetite, sleep, and pain, significantly improved throughout the study. No significant adverse effects were reported, indicating that pyrroloquinoline quinone is safe for consumption over an eight-week period. These findings highlight the potential of pyrroloquinoline quinone as a dietary supplement for improving mental and physical health, especially in individuals facing stress and sleep disorders [209].

## 8. What Is Needed for Biocompounds to Be Used as Medications?

For a natural molecule to be considered a drug, it must undergo a series of rigorous processes, which can be divided into several stages, from initial identification to regulatory approval. Each of these stages can take years and requires a solid scientific, technical, and regulatory foundation, along with significant financial investment. A small fraction of the molecules that enter clinical trials ultimately reach the market as approved drugs. Currently, research in the Amazon region focuses on screening, isolating, and purifying compounds from plant extracts or fractions [210,211].

Once the feasibility of obtaining, characterizing, and purifying the compound is verified, biological, and/or pharmacological activity tests begin. In in vitro studies, the compound is tested in cell cultures to assess its biological efficacy, such as inhibiting cell growth, modulating metabolic pathways, and evaluating toxicity. If the molecule shows promising results in vitro, it is tested in animal models to assess toxicity, effective dose, and pharmacokinetics (how the molecule is absorbed, distributed, metabolized, and excreted by the organism). Additionally, short- and long-term toxicological tests are conducted, such as genotoxicity, teratogenicity, and carcinogenicity assessments [11,12,13].

If the evidence remains favorable, clinical trials are conducted in three phases (I, II, and III), escalating in participant numbers, types of interventions, and comparisons with existing drugs. Following successful clinical trials, the results are compiled into a dossier that is submitted to regulatory agencies (such as the Food and Drugs Administration [FDA] in the United States of America, Agência Nacional de Vigilância Sanitária [ANVISA] in Brazil, or European Medicines Agency [EMA] in Europe). If approved, the molecule can be marketed as a drug, with pharmacovigilance monitoring to identify any rare or long-term adverse effects in a larger population [211].

For biocompounds to be used as medications, several challenges must be overcome during the research, development, and regulatory phases. Unfortunately, research on Amazonian biocompounds is often conducted on a small scale, frequently limited to in vitro approaches. To reach the clinical trial stage, it is necessary to scale up compound production without compromising its efficacy or environmental sustainability—this requires adequate infrastructure, funding, and partnerships with research institutions and pharmaceutical companies.

Many natural compounds, despite their promise, have low bioavailability, meaning they are not easily absorbed or metabolized in the human body. The composition of plant extracts can vary significantly depending on factors such as season, harvest region, and extraction methods. Therefore, it is essential to standardize extraction and formulation methods to ensure consistency in the drug.

Obtaining patents for natural biocompounds is a complex process, as many of these substances are already known to traditional communities. Government programs and policies that incentivize biotechnology can accelerate the research and development of medications derived from Amazonian biocompounds. These steps are crucial for transforming the vast therapeutic potential of Amazonian biocompounds into effective and safe medications that can benefit human health in a sustainable and ethical manner.

## 9. Conclusions

The biodiversity of the Amazon offers substantial potential for discovering new molecules with therapeutic properties through various in vitro approaches. Many plants from the region have been traditionally used by traditional communities to treat a range of illness, including parasitic diseases, or as prophylactics, and these traditional uses can serve as guidance for developing new drugs. Amazonian biocompounds present a diverse array of pharmacological properties with substantial therapeutic potential, encompassing antimicrobial, anti-inflammatory, and antineoplastic activities.

These compounds exert their effects through various mechanisms, such as disrupting cell membranes and modulating microbial metabolism. Phenolic compounds provide cardiovascular and neuroprotective benefits, while others exhibit potent anti-inflammatory properties and support antioxidant mechanisms. Sesquiterpenes derived from specific plants demonstrate efficacy against protozoans with minimal toxicity, whereas, a range of alkaloid-rich extracts exhibit notable antiparasitic activity. Additionally, polysaccharides from select plants display cytostatic effects on cancer cells, with sesquiterpenes and terpenes inducing apoptosis and influencing angiogenesis. Advances in nanostructured lipid carriers and molecular modifications are enhancing the delivery and therapeutic efficacy of these bioactive compounds, underscoring their potential in pharmaceutical applications.

## Figures and Tables

**Table 1:** Summary of the information present in the selected articles in relation to the method of obtaining metabolites, major compounds, techniques for measuring usability, safety, and biological activity.

	Major Compound(s)	Plant Group/Species	Type of Extraction	Technique(s) Used to Measure Biological Activity	Biological Activity	Ref.
**MULTIPLE PHARMACEUTICAL PROPERTIES**	Phenolic compounds	*Caryocar villosum*	Ethanolic and hydroethanolic extract	DPPH; ABTS; DCF-DA; alamar blue assay; NO assay; hemolytic assay	Anti-inflammatory; antineoplastic; antioxidant activity	[24]
	Phenolic compounds	*Couroupita guianensis*	Decoction	MTT; migration assay; Western blot	Anti-inflammatory; cicatrization	[25]
	Phenolic compounds	*Astrocaryum aculeatum*	Hydroethanolic extract	MTT; flow cytometry; lipid and protein oxidation; SOD analysis; RT-PCR	Anti-inflammatory; decrease ROS species and increase in antioxidant defense; positive regulation of the cell cycle	[26]
	Phenolic compounds	*Euterpe oleracea*	Methanol extract; liquid chromatography	Cell viability with DAPI; comet assay; FRAP	Antimicrobial; cytotoxicity; possible antigenotoxic effect	[27]
	Phenolic compounds	*Libidibia ferrea*	Hydroethanolic extract	MTT; apoptosis/necrosis test; CBMN; comet assay; cell migration	Antioxidant activity; antineoplastic	[28]
	Terpenes	*Carapa guianensis*	Seed oil; silica gel column chromatography	Cell viability (MTT); NO assay	Anti-inflammatory	[29]
	Terpenes	*Croton cajucara*	Soxhlet; molecular modifications	Clonogenic assay; apoptosis/necrosis test, comet assay; CBMN	Modified molecules decreased cytogenotoxicity	[30]
**ANTIPARASITIC ACTIVITY**	Phenolic compounds	*Eugenia* sp.	Hydroethanolic extract; dichloromethanolic fraction; hydroalcoholic residue	MTT; DPPH; hematotoxicity	Anthelmintic	[31]
	Phenolic compounds	*Equisetum hyemale*	Hydroalcoholic extract; acetate, dichloromethanolic, and n-butanolic fractions	Cell viability (MTT); Comet Assay	Antimicrobial	[32]
	Phenolic compounds	*Deguelia nitidula*	Ethanol extraction	MTT; antibacterial bioassay	Antimicrobial	[33]
	Phenolic compounds; steroids	*Abuta grandiflora*; *Ambelania duckei*; *Aspidosperma excelsium*; *Curarea toxicofera*	Aqueous percolation	Resazurin assay	Anti-*Trypanosoma cruzi* activity	[34]
	Terpenes	*Ocotea* sp.	Hydrodistillation to obtain essential oil	MTT	Antimicrobial	[35]
	Terpenes	*Copaifera reticulata*	Manual extraction of oleoresins	MTT; anti-*Plasmodium* test in erythrocytes	Anti-*Plasmodium* activity	[36]
**ANTINEOPLASTIC PROFILE**	Phenolic compounds	*Portulaca* sp.	Aqueous and hydroalcoholic sonication	MTT; flow cytometry	Antineoplastic against colorectal adenocarcinoma	[37]
	Fatty acids	*Carapa guianensis*	Extraction handcrafted with organic solvent	MTT; apoptosis/necrosis test; CBMN	Antineoplastic against gastric adenocarcinoma	[38]
	Terpenes	Seven species from Myrtaceae family	Hydrodistillation to obtain essential oil	MTT	Antineoplastic against melanoma, gastric and colon cancer	[39]
	Terpenes	*Eugenia* sp.	Hydrodistillation to obtain essential oil	MTT	Antineoplastic against colon cancer	[40]
	Terpenes	*Croton cajucara*	Hydrodistillation	Reazurin assay; DPPH; DCF-DA	Antineoplastic against basal alveolar adenocarcinoma	[41]
**COSMECEUTICAL OR NUTRACEUTICAL**	Phenolic compounds; fatty acids	*Euterpe oleracea*	Patent protected method	MTT; CBMN; comet assay	Absence of cytogenotoxicity	[42]
	Quinones	*Eleutherine plicata*	Chromatographic column	*Allium cepa* assay; CBMN	Genotoxicity	[43]
	Fatty acids; phenolic compounds	*Byrsonima crassifolia*	Extraction with supercritical CO_2_	MTT	Cytoprotectivity	[44]
	Phenolic compounds	*Copaifera malmei*	Aqueous infusion	CBMN; comet assay	Antigenotoxic	[45]
	Fatty acids	*Carapa guianensis*	Pressing dry seeds; heat treatment and Soxhlet extraction	DPPH; Ames test; CBMN	Cytogenotoxicity	[46]
	Quinones	*Eleutherine plicata*	Ethanol extraction; dichloromethanolic fraction	MTT; comet assay	Cytogenotoxicity	[47]
	Fatty acids	*Carapa guianensis*	Oil and nanoemulsion	MTT; CBMN; comet assay	Cytotoxicity	[48]

Legend: ABTS = 2,2-azinobis (3-ethylbenzothiazoline-6-sulfonic acid); CBMN = micronucleus test with cytokinesis block; CO_2_ = carbon dioxide; DAPI = 4’,6-diamidino-2-phenylindole; DCF-DA = 2′,7′-dichlorofluorescin diacetate; DPPH = 2,2-diphenyl-1-picrylhydrazyl; FRAP = ferric reducing antioxidant power; MTT = tetrazoline 3-(4,5-dimethylthiazol-2yl)-2,5-diphenyl bromide; NO = nitric oxide; ROS = reactive oxygen species; RT-PCR = real-time polymerase chain reaction; SOD = superoxide dismutase.

## Supplementary Materials

The following supporting information can be downloaded at: https://www.mdpi.com/article/10.3390/ph17111449/s1, Figure S1: Outline of the active search and selection of articles to compose the literature review; Table S1: Inclusion and exclusion criteria for selecting in vitro studies assessing the efficacy and safety of plant metabolites from the Amazon region.
