# Supplementary material for: Prospecting Pharmacologically Active Biocompounds from the Amazon Rainforest: In Vitro Approaches, Mechanisms of Action Based on Chemical Structure, and Perspectives on Human Therapeutic Use
Source: Pharmaceuticals (Basel). 2024 Oct 30;17(11):1449. doi: 10.3390/ph17111449 (PMC11597570; doi:10.3390/ph17111449)

The literature review to search for research on pharmacologically active biocompounds from the Amazon region. was carried out manually in the main virtual repositories available online, namely: ScienceDirect, Pubmed and Scielo. The keywords used were "amazon", "plant", "vegetable", "*in vitro*" and "metabolite" and plural, combined or not, in addition to the Boolean operators "AND" and "OR" to refine the results. In relation to filters, we limited the time period between the years 2017 to 2023 and exclusively articles written in English. The inclusion and exclusion criteria (Table S1) to define the parity of the bibliography returned from the databases with the theme proposed for this review were previously delimited.

Table S1 [Supplementary Data]. Inclusion and exclusion criteria for selecting in vitro studies assessing the efficacy and safety of plant metabolites from the Amazon region.

Inclusion criteria.	Exclusion criteria
In vitro studies	*In vivo* studies
Studies investigating biological activity of plant metabolites	Studies that evaluate metabolites from other living beings
Studies after 2017	Studies prior to 2017
Studies that characterize at least the majority metabolites	Studies without proper phytochemical characterization
Studies published in peer-reviewed journals	Studies in summary form, reviews, dissertations, and theses
Open access articles	Articles with restricted access

The filtering and selection of articles were conducted as follows: first the title and summary of the works were read and those that already reported some exclusion criteria were soon disregarded. Screening was carried out on the pre-selected articles to attest to the quality of the evidence attested by the authors. Finally, after a thorough analysis of the content of the articles and their appropriate adjustments to the inclusion and exclusion criteria, those that make up this review were selected. The entire process mentioned above was done independently by authors AOAV and OVMS, and an alignment meeting was subsequently held to actually decide which articles would undergo screening and be added to the discussion of this review. The process is illustrated in Figure 1.

Figure S1. [Supplementary Data]. Outline of the active search and selection of articles to compose the literature review.

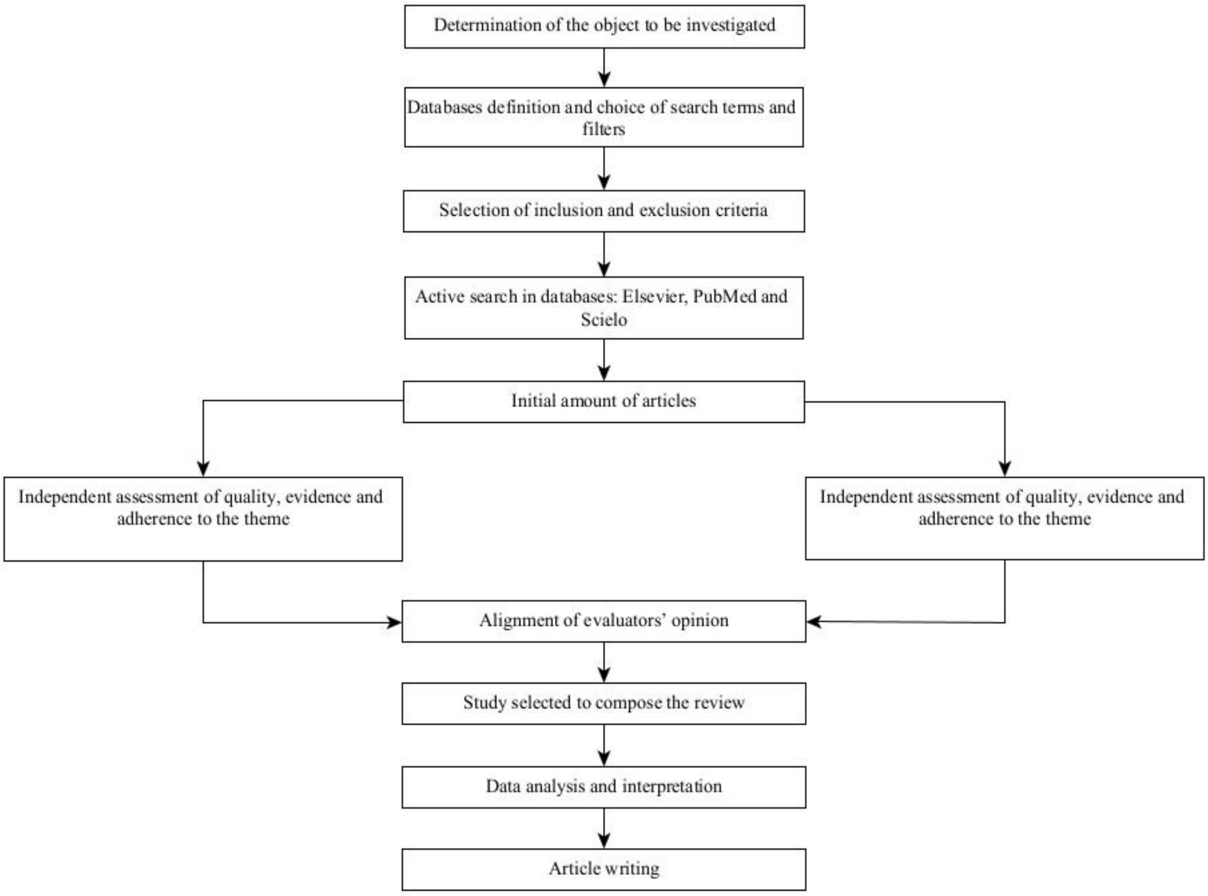

Supplement: Supplementary file 1 [file pharmaceuticals-17-01449-s001.zip › pharmaceuticals-3211624-supplementary.pdf]
